# Prévalence du trait drépanocytaire chez les donneurs de sang au centre régional de transfusion sanguine de la région Haute Matsiatra, Madagascar

**DOI:** 10.11604/pamj.2020.36.329.21478

**Published:** 2020-08-24

**Authors:** Jocia Fenomanana, Irène Rakotoniaina, Stéphania Niry Manantsoa, Harinirina Randriamahenina, Zely Arivelo Randriamanantany

**Affiliations:** 1 Service Laboratoire, CHU Andrainjato, Fianarantsoa, Madagascar,; 2 Service Laboratoire, CHU Mitsinjo Betanimena, Tuléar, Madagascar,; 3 Service Laboratoire, CHU-JRA, Tananarive, Madagascar,; 4 Direction de la Transfusion Sanguine, Tananarive, Madagascar

**Keywords:** Donneurs de sang, drépanocytose, Madagascar, Blood donors, sickle cell disease, Madagascar

## Abstract

**Introduction:**

le trait drépanocytaire correspond à la forme hétérozygote de la drépanocytose. Les porteurs du trait drépanocytaire sont capables de synthétiser de l´hémoglobine A normale et de l´hémoglobine S. Cette affection est cliniquement inapparente de telle sorte que des porteurs du trait drépanocytaire, ignorant leurs statuts génétiques, peuvent se retrouver parmi les donneurs de sang. Cette situation peut avoir des conséquences sévères sur leurs propres états de santé ainsi qu´aux receveurs, particulièrement si ces derniers sont drépanocytaires. L´objectif de notre étude est de déterminer la prévalence du trait drépanocytaire chez les donneurs de sang.

**Méthodes:**

nous avons réalisé une étude prospective descriptive sur 4 mois (de janvier à mai 2017) au Centre Régional de Transfusion Sanguine (CRTS) Haute Matsiatra. Tous les donneurs ont été dépistés par test d´Emmel et confirmation des cas positifs par électrophorèse de l´hémoglobine.

**Résultats:**

nous avons recruté 427 donneurs dont 332 hommes et 95 femmes avec un sex-ratio de 3,4. L´âge moyen des donneurs était de 32,72 ans allant de 18 à 64 ans. Le test d´Emmel s´est révélé positif chez 5 donneurs (1,17%), tous confirmés de génotype AS à l´électrophorèse de l´hémoglobine.

**Conclusion:**

les résultats de cette étude révèlent l´existence de porteurs de trait drépanocytaire parmi les donneurs de sang du CRTS dont la plupart ignore leur statut drépanocytaire avant le don de sang. Afin d´assurer la sécurité transfusionnelle et la qualité des produits sanguins, le dépistage de la drépanocytose chez les donneurs de sang est désormais une question pertinente et d´actualité.

## Abstract (en)

**Introduction:**

sickle cell trait is the heterozygous form of sickle-cell disease. Patients with sickle cell trait can synthesize normal hemoglobin A and hemoglobin S. This condition has no recognizable clinical signs; then subjects with sickle cell trait, ignoring their genetic status, can be found among blood donors. This can have severe impact on donors´ health status and on that of recipients, especially if these have sickle-cell trait. The purpose of our study is to determine the prevalence of sickle cell trait in blood donors.

**Methods:**

we conducted a 4-month descriptive prospective study (January-May 2017) at the Haute Matsiatra Regional Blood Transfusion Center (RBTC). All donors were screened by Emmel test and positive cases were confirmed by hemoglobin electrophoresis.

**Results:**

the study involved 427 donors, of whom 332 were men and 95 women (sex ratio 3.4). The average age of blood donors was 32.72, ranging from 18 to 64 years. Emmel test was positive in 5 donors (1.17%). These patients had the AS genotype confirmed by hemoglobin electrophoresis.

**Conclusion:**

the results of this study reveal the presence of sickle cell trait among blood donors at the CRTS. Most of them ignore their sickle cell status before blood donation. Quality and safety of blood and blood products are mandatory, hence the importance of screening among blood donors is a current relevant issue.

## Introduction

La drépanocytose est une maladie génétique de l´hémoglobine à transmission autosomique récessive. Elle est due à la mutation du 17^e^ nucléotide (T-A) du gène de la β globine, provoquant la substitution de l´acide glutamique en valine au niveau du 6^e^ AA du segment A de la chaîne. Il en résulte la présence de l´hémoglobine S (Hb S) dans les globules rouges qui se déforment en faucilles (drépanocytes) rigides [1-3]. Ces derniers sont responsables de l´obstruction de veinules post capillaires, d´infarctus dans divers organes, de la libération de molécules pro-inflammatoires, de l´expression accrue de molécules d´adhésion, d´une hypercoagulabilité, et d´une hémolyse secondaire des drépanocytes [4,5]. La drépanocytose est une pathologie fréquente [1,2] observée principalement chez les sujets noirs d´Afrique équatoriale, mais également dans le pourtour méditerranéen, le Moyen Orient, le continent indien et les zones de migration de ces populations (Amérique du nord et du sud). Sa prévalence globale à Madagascar est estimée à 11%. Cette prévalence est plus élevée dans la Région du Sud Est, de forte endémicité, mais la maladie intéresse tout le territoire à cause du brassage de la population [3]. Dans sa forme homozygote, les symptômes de la drépanocytose apparaissent vers le 6^e^ mois de la vie, avec des signes d´anémie hémolytique chronique avec un syndrome anémique, un ictère, une splénomégalie, un retard de croissance chez l´enfant en bas âge. Les complications aigües sont dominées par les crises vaso-occlusives douloureuses abdominales ou osseuses. Les complications chroniques sont représentées par une asplénie fonctionnelle, des atteintes cardio-respiratoires ou rénales, une ostéonecrose de la tête fémorale ou un ulcère de jambe [5,6]. Cependant, chez le sujet hétérozygote, la maladie est généralement asymptomatique [7-9].

Le traitement transfusionnel reste un élément majeur de la prise en charge thérapeutique de la drépanocytose [10,11]. Un approvisionnement suffisant et sécurisé en sang devrait faire partie intégrante de toute politique nationale de santé et de l´infrastructure sanitaire d´un pays. Selon l´OMS, près de 112.5 millions de dons de sang sont collectés chaque année dans le monde [12,13]. Toutefois, avec une prévalence de 11% de la drépanocytose dans la population générale à Madagascar, des porteurs du trait drépanocytaire peuvent, sans le savoir, se retrouver parmi les donneurs de sang volontaires puisque la majorité est asymptomatique [14-16]. Cette situation peut avoir des conséquences sévères chez les receveurs particulièrement si ces derniers sont drépanocytaires (S/S). En effet, la transfusion de sang contenant de l´hémoglobine S à un sujet drépanocytaire va augmenter secondairement la proportion des hématies falciformes déjà présentes dans la circulation et va favoriser encore plus la formation d´occlusions dans la microcirculation. Cette augmentation de la proportion d´hémoglobine S va priver les tissus et les organes d´oxygène, contradictoirement à l´effet attendu d´une transfusion de sang normal [5,13]. De plus, la transfusion de sang contenant de l´hémoglobine S ne donne pas les mêmes effets thérapeutiques qu´une poche de sang normale, à cause du problème de déleucocytation pendant la phase de préparation des produits sanguins et de la diminution de la déformabilité des rouges [17-20]. Les études effectuées pour déterminer la fréquence de cette hémoglobinopathie sont rares chez les donneurs de sang. Ainsi, l´objectif général de notre étude est de déterminer la prévalence du trait drépanocytaire parmi les donneurs de sang.

## Méthodes

Cette étude a été réalisée au sein du Centre Régional de Transfusion Sanguine (CRTS) de Haute Matsiatra sis à Fianarantsoa, une ville située à 400km de la capitale de Madagascar. Il s´agit d´une étude prospective descriptive d´une durée de cinq mois allant du mois de janvier au mois de mai 2017. Tous les donneurs de sang, quel que soit leurs statuts (donneur familial/de remplacement, donneur bénévole régulier) ont été inclus. Prélèvements: environ 2 ml de sang veineux est recueilli sur tube EDTA pour chaque donneur. Ce prélèvement est réalisé à partir de la tubulure de la poche de sang, une fois que le prélèvement pour la poche ait été terminé. Pour chaque échantillon, un test d´Emmel a été effectué et confirmation par électrophorèse de l´hémoglobine des cas positifs.

La réalisation du test se fait sur lame porte-objet, 50μl de sang est mélangé à 50µl de métabisulfite de sodium à 2%. Le tout est recouvert de lamelle couvre objet, puis scellé avec de la paraffine. La lecture et l´interprétation se font après 24 heures d´incubation à température ambiante, sur microscope optique /objectif x40. Un contrôle positif et négatif sont traités avec chaque série de test. Le test est positif s´il y a transformation des hématies en forme de « faucille ». Il est négatif si les hématies gardent leurs formes normales après 48h [21-24]. Electrophorèse des hémoglobines Nous avons utilisé comme réactif le kit SEBIA Hydragel Hemoglobin 7/15 [25]. Il s´agit d´une électrophorèse à pH alcalin sur gel d´acetate de cellulose. Elle a été réalisée sur l´automate Hydragel SEBIA®.

## Résultats

Au total, durant les cinq mois d´étude, nous avons inclus 427 donneurs de sang, âgés de 18 à 64 ans, répartis de la manière suivante. L´âge moyen des donneurs était de 32,72 ± 11,46 ans avec des extrêmes de 18 et 64 ans. Le groupe d´âge de 18 à 30 ans était le plus représenté (46,60%) avec une prédominance masculine donnant un sex-ratio de 3,4 (Tableau 1). Tous les donneurs inclus dans cette étude ignoraient leurs statuts drépanocytaires au moment du don et 38,40% des donneurs ont déjà effectué un don de sang auparavant. Parmi les 427 donneurs, 366 soit 85,71% était des donneurs familiaux, 8,19% de donneurs bénévoles réguliers, 6,08% de nouveaux donneurs bénévoles, et aucun donneur rémunéré. Le test d´Emmel était positif chez 5 donneurs de sang soit 1,17% (3 hommes et 2 femmes), tous confirmé comme étant hétérozygote AS à l´électrophorèse de l´hémoglobine (Tableau 2).

**Tableau 1:** caractéristiques démographiques des donneurs de sang

Variables	Nombre	Pourcentage (%)
**Genre**		
Masculin	332	77,28
Féminin	95	22,72
Total	427	100
**Groupe d´âge**		
[18-30[	199	46,60
[30-45[	147	34,42
[45-60[	76	17,80
[60-64[	5	1,18
Total	427	100
**Connaissance du statut drépanocytaire**		
Oui	0	0
Non	427	100
Total	427	100
**Don de sang antérieur**		
Oui	164	38,40
Non	263	61,60
Total	427	100

**Tableau 2:** proportion des donneurs selon le genre et les résultats de dépistage du trait drépanocytaire A/S

	Test d´Emmel		Génotype AS
Genre	Négatif	Positif
Masculin	327 (99,09%)	3 (0,91%)	3 (0,91%)
Féminin	95 (97,94%)	2 (2,06%)	2 (2,06%)
Total	422(98,83%)	5 (1,17%)	5 (1,17%)

## Discussion

La transfusion sanguine est une procédure thérapeutique qui sauve de nombreuses vies humaines mais qui peut aussi être responsable de complications graves. Chaque don de sang est susceptible d´engendrer des risques potentiels tant chez le donneur que chez le receveur [26,27]. Certains individus présentant une pathologie quelconque peuvent être retrouvés parmi les donneurs de sang s´ils sont asymptomatiques. Un approvisionnement suffisant et surtout sécurisé en sang devrait faire partie intégrante de toute politique nationale de santé et de l´infrastructure sanitaire d´un pays. Dans cette étude, une prédominance masculine (77,28%) a été observée chez les donneurs avec un sex-ratio de 3,4. Ce résultat rejoint celui d´une étude où le ratio et de 12,1 [20,28]. Cette situation peut être attribuée au fait que les femmes ne sont pas souvent encouragées à se porter volontaire au don de sang à cause de certaines croyances socio-culturelles. De plus, certaines conditions physiologiques féminines telles que la grossesse, la lactation et menstruation sont des contre-indications au don de sang [29]. La distribution selon l´âge montre que le groupe de 18-30 ans est le plus représenté (46,6%), ce qui est similaire aux résultats d´Omisakin *et al*. [20]. La totalité (100%) des participants ignoraient leur statut génétique vis-à-vis de l´hémoglobinose S, similairement à l´étude de Lippi *et al*. [29]. En effet, les sujets porteurs de trait AS sont généralement asymptomatiques, les paramètres hématologiques (hémoglobine, indices érythrocytaires) sont souvent normaux [30]. La prévalence du trait AS de 1,17% retrouvée dans cette étude est significative pour un centre de transfusion. D´autres études par Omisakin *et al*. et Zaccheaus *et al*. ont retrouvé des prévalences respectives de 26,1% et 19,68% [20,31].

Par ailleurs, l´OMS encourage tous les pays à se doter de services de transfusion reposant intégralement sur le don de sang volontaire et non rémunéré. Ces derniers sont également le groupe de donneurs le plus sûr car la prévalence des infections véhiculées par le sang y est la plus faible [31]. Pourtant dans cette étude, seulement 8,19% parmi les donneurs étaient des volontaires contre 85,71% de donneurs de remplacement. Il est primordial de détecter le trait drépanocytaire chez les futurs donneurs bénévoles afin de préserver leur état de santé et celui des receveurs.

## Conclusion

On en déduit qu´il y a un nombre significatif de porteur de trait drépanocytaire parmi les donneurs de sang du CRTS Haute Matsiatra. Ces derniers ignorent leur statut génétique vis-à-vis de la drépanocytose et les risques que leurs dons pourraient causer à certains patients receveurs. Un dépistage systématique du trait AS avant le don de sang ou dans les poches de sang s´avère être une question pertinente et d´actualité. Grace à ce procédé, le risque de transfuser du sang contenant l´hémoglobine S chez un drépanocytaire pourrait être évité, un pas de plus vers l´amélioration de la prise en charge de la drépanocytose.

### Etat des connaissances sur le sujet

- Le trait drépanocytaire est souvent asymptomatique;
- La transfusion de sang contenant de l´hémoglobine S peut avoir des conséquences sévères chez les receveurs particulièrement si ces derniers sont drépanocytaires (S/S), au risque d´augmenter secondairement la proportion des hématies falciformes et de favoriser la formation d´occlusions dans la microcirculation.

### Contribution de notre étude à la connaissance

- Des porteurs du trait drépanocytaire peuvent être retrouvés parmi les donneurs de sang car ils sont souvent asymptomatiques;
- Le dépistage systématique de la drépanocytose chez les donneurs de sang, en zones d´endémie drépanocytaire, est indispensable pour éviter de transfuser du sang contenant de l´hémoglobine S chez les receveurs, particulièrement si ces derniers sont drépanocytaires homozygotes.
